# Transcriptomic Changes Drive Physiological Responses to Progressive Drought Stress and Rehydration in Tomato

**DOI:** 10.3389/fpls.2016.00371

**Published:** 2016-03-31

**Authors:** Paolo Iovieno, Paola Punzo, Gianpiero Guida, Carmela Mistretta, Michael J. Van Oosten, Roberta Nurcato, Hamed Bostan, Chiara Colantuono, Antonello Costa, Paolo Bagnaresi, Maria L. Chiusano, Rossella Albrizio, Pasquale Giorio, Giorgia Batelli, Stefania Grillo

**Affiliations:** ^1^National Research Council of Italy, Institute of Biosciences and Bioresources, Research Division Portici (CNR-IBBR)Portici, Italy; ^2^National Research Council of Italy, Institute for Agricultural and Forestry Systems in the Mediterranean (CNR-ISAFoM)Ercolano, Italy; ^3^Department of Agriculture, University of Naples “Federico II,”Portici, Italy; ^4^CREA - Council for Agricultural Research and Economics, Genomics Research CentreFiorenzuola d'Arda, Italy

**Keywords:** ABA, gene-expression cluster analysis, photosynthesis, proline, RNA sequencing, stomatal conductance, water stress

## Abstract

Tomato is a major crop in the Mediterranean basin, where the cultivation in the open field is often vulnerable to drought. In order to adapt and survive to naturally occurring cycles of drought stress and recovery, plants employ a coordinated array of physiological, biochemical, and molecular responses. Transcriptomic studies on tomato responses to drought and subsequent recovery are few in number. As the search for novel traits to improve the genetic tolerance to drought increases, a better understanding of these responses is required. To address this need we designed a study in which we induced two cycles of prolonged drought stress and a single recovery by rewatering in tomato. In order to dissect the complexity of plant responses to drought, we analyzed the physiological responses (stomatal conductance, CO_2_ assimilation, and chlorophyll fluorescence), abscisic acid (ABA), and proline contents. In addition to the physiological and metabolite assays, we generated transcriptomes for multiple points during the stress and recovery cycles. Cluster analysis of differentially expressed genes (DEGs) between the conditions has revealed potential novel components in stress response. The observed reduction in leaf gas exchanges and efficiency of the photosystem PSII was concomitant with a general down-regulation of genes belonging to the photosynthesis, light harvesting, and photosystem I and II category induced by drought stress. Gene ontology (GO) categories such as cell proliferation and cell cycle were also significantly enriched in the down-regulated fraction of genes upon drought stress, which may contribute to explain the observed growth reduction. Several histone variants were also repressed during drought stress, indicating that chromatin associated processes are also affected by drought. As expected, ABA accumulated after prolonged water deficit, driving the observed enrichment of stress related GOs in the up-regulated gene fractions, which included transcripts putatively involved in stomatal movements. This transcriptomic study has yielded promising candidate genes that merit further functional studies to confirm their involvement in drought tolerance and recovery. Together, our results contribute to a better understanding of the coordinated responses taking place under drought stress and recovery in adult plants of tomato.

## Introduction

Drought conditions historically constitute the abiotic stress with the biggest impact on crop yield and agricultural productivity (Boyer, 1982; Boyer et al., 2013). Predicted climate change threatens stable crop yields, will likely require changes in agricultural practices in response to altered rainfall patterns, and increased urban consumption. Multiple projections indicate that between 20 and 60 Mha of irrigated cropland may have to be reverted to rainfed management by the end of the century (Elliott et al., 2014). Formerly irrigated crops would become entirely dependent on rainfall and vulnerable to yield loss due to drought. This reduction is predicted to be severe in southern Spain and Italy, two major producers of tomatoes (Saadi et al., 2015). This increasing vulnerability to drought requires that we develop more resilient varieties capable of surviving drought conditions while maintaining yield (Mickelbart et al., 2015). Both traditional breeding and targeted genome editing for drought tolerance require a better understanding of drought responses mechanisms (Langridge and Reynolds, 2015), which include molecular mechanisms governing the timing of stomata closure, modulation of photosynthetic performances, accumulation of osmolytes, and growth retardation (Bosco De Oliveira et al., 2012). Stomata represent the first barrier plants employ to avoid dehydration, with the trade-off of a reduced CO_2_ supply to the mesophyll. As water deficit intensifies, metabolic impairments impose further limitations to photosynthesis (Chaves et al., 2009). Along with plant responses to water deficit, understanding the recovery of photosynthesis upon rehydration is of paramount importance to understand water stress effects on photosynthesis (Flexas et al., 2004).

Impact of drought on gene expression has been intensely analyzed in numerous species such as Arabidopsis (Sakuraba et al., 2015), rice (Oono et al., 2014), maize (Kakumanu et al., 2012), sorghum (Dugas et al., 2011), and poplar (Barghini et al., 2015) by high-throughput transcriptomics.

A predominant role in driving drought-induced changes in gene expression is played by the hormone abscisic acid (ABA). The mechanisms of ABA perception and signal transduction are the subject of intense research and major breakthroughs have included the identification of a family of cellular receptors (Ma et al., 2009; Park et al., 2009). An increase in ABA changes the hydraulic regulation of stomata (Chaves et al., 2009), resulting in stomata closure under adverse hydraulic conditions by controlling guard cells behavior and decreasing water permeability within the leaf vascular tissue (Pantin et al., 2013). Tomato (*Solanum lycopersicum* L.) is one of the major horticultural crops and an important dietary source of vitamins A and C as well as carotenoids such as lycopene (Canene-Adams et al., 2005). Tomato is also considered a plant model system for fleshy fruit development (Osorio et al., 2011) and interaction with pathogens (Andolfo and Ercolano, 2015), with several tools available, including the sequenced genome (Sato et al., 2012) and its large open source genomic repository (Suresh et al., 2014). Although tomato is cultivated worldwide, it is considered sensitive to stresses of biotic and abiotic nature (Rai et al., 2013; Kissoudis et al., 2015). Most modern tomato cultivars are sensitive to water deficit, which results in reduced seed development and germination, reduced vegetative growth, and impaired reproduction (Nuruddin et al., 2003; Bartels and Sunkar, 2005; Rai et al., 2013). Galmés et al. (2011) reported that Mediterranean drought-tolerant tomato showed higher intrinsic water use efficiency under water stress compared to a tomato accession originating from a humid habitat. Such results could be due to stress induced changes in anatomical development (Galmés et al., 2013) or the abundance and activity of aquaporins facilitating CO_2_ transport through the mesophyll (Kaldenhoff, 2012).

The average water footprint per Kg of tomato is 215 liters, 30% of which is supplied by irrigation (Mekonnen and Hoekstra, 2011). Therefore, it is essential to develop drought tolerant, higher yielding varieties to cope with the increasing demand for tomato (Solankey et al., 2014). Only limited mapping research has been conducted on drought tolerance compared to other abiotic stresses in tomato (Foolad, 2007). Wild relatives, such as the drought tolerant *Solanum pennellii*, represent a valuable source of novel traits for genetic improvement. Gene expression studies in response to water stress have been carried out in leaves using microarray approaches identifying differentially expressed transcripts of genes involved in energy, plant hormones biosynthesis, and cation transporters and a number of transcription factors and signaling proteins (Gong et al., 2010; Sadder et al., 2014). However, studies that integrate the different levels of response to drought stress in tomato are under-represented.

The goal of our research was to identify in tomato the transcriptomic changes that control physiological adjustments under drought stress and recovery. In this study, we subjected plants to two drought treatments separated by a rehydration and recovery phase. We performed RNA sequencing on each of these stages to establish transcriptomes for drought and recovery. Each transcriptomic set is accompanied by physiological measurements (chlorophyll fluorescence, CO_2_ assimilation, and stomatal conductance), quantification of key metabolites (proline and ABA), and biometric parameters. These measurements serve to define the conditions of drought and recovery and provide a snapshot of the physiological state in each condition. We performed genome-wide comparisons of transcriptomes using cluster analysis to identify stress-induced patterns of expression and integrated them with physiological responses.

## Materials and methods

### Plant materials, growth conditions, and stress treatments

Seeds of cultivar M82 (accession LA3475) supplied by the Tomato Genetics Resource Center (TGRC, http://tgrc.ucdavis.edu/) were germinated in soil in a semi-controlled greenhouse. Air temperature (T_a_, °C), humidity (RH, %), and solar radiation (R_s_, W m^−2^) were acquired by a data logger (Spectrum Technologies, Plainfield, IL). The average air humidity and temperature were 58% and 22°C during the day and 86% and 18°C during night-time, while the cumulated daily global radiation during the day was 8.4 (±3.6) MJ/m^2^ day.

When seedlings had developed two true leaves (25 days after sowing seeds), they were transplanted in pots, filled with soil (one plant per pot) and fertilized after 7 days with Nitrophoska gold (Compo Agricoltura, Cesano Maderno, Italy). Plants were well irrigated for 30 days prior to start the stress treatments. Then plants were equally divided into control and stress treatment, nine replicates per treatment, and arranged in a randomized block design.

Two cycles of water deficit were performed by water withholding until soil water content of stress pots was < 1/3 of control pots, inducing a nearly complete closure of the stomata. This corresponded to 16 and 6 days of water withholding in the first and second cycle of drought, respectively. Between these two stress cycles, plants were well irrigated allowing a full recovery of soil water content and stomatal conductance. Control plants were well watered throughout the entire experimental period.

During the experiment, the soil water content (θ, m^3^/m^3^) was determined from dielectric measurements performed by a Time Domain Reflectometer (TDR100 Campbell Scientific Inc. Logan, UT) and applying the Topp's equation. The 14.2 cm trifilar probes were placed in 3 pots per treatment.

Leaf samples for molecular and biochemical analyses were collected at different time point of the experiment.

### Gas exchange and modulated Chl *a* fluorescence emission

Net photosynthetic CO_2_ assimilation rate (A, μmol m^−2^ s^−1^) and stomatal conductance to water vapor (g_s_, mol m^−2^ s^−1^) were measured on a fully expanded, well-exposed top leaf on 5–6 plants per treatment between 10:00 a.m. and 1:00 p.m. Measurements were carried out using a portable open-system gas-exchange and modulated fluorometer analyser Li-6400XT (Li-Cor Biosciences, Lincoln, NE, USA), with CO_2_ inside leaf chamber set to 400 μmol CO_2_ mol^−1^ air. An artificial light source LED with emission peaks centered at 635 nm in the red and at 465 nm in the blue provided a PPFD equal to 2000 μmol (photons) m^−2^ s^−1^ (90% red, 10% blue). The instrument was also used to measure the steady-state (F′) and, upon a 0.8 s saturating light pulse emission, the maximum (F${}_{\text{m}}^{\prime }$) Chl *a* fluorescence emission of leaves under actinic light. The software of the instrument (Li-Cor, 2011) calculated the gas-exchange parameters on the basis of von Caemmerer and Farquhar (1981) model, and the effective quantum yield of PSII photochemistry in light-adapted leaves, Φ_PSII_ = ($F_{\text{m}}\prime$– F′)/$F_{\text{m}}\prime$ according to Genty et al. (1989).

### Transient Chl *a* fluorescence emission

For fluorescence assays a continuous excitation Handy PEA fluorometer (Hansatech, Instruments Ltd., King's Lynn, Norfolk, England) was used. The excitation red light pulse for fluorescence induction (FI) was emitted by a (red) 650 nm light diode source, and applied for 1 s at the maximal available (sub-saturating) photosynthetic photon flux density (PPFD) of 3500 μmol (photon)/m^2^ s. Leaves were dark adapted for 30 min by means of the equipped white leaf-clips, prior to the assessment of the basal fluorescence emission (F_o_), and the peak fluorescence emission (F_p_) reached during the fast polyphasic raise induced by the sub-saturating excitation light pulse. As F_p_ is a viable approximation of the maximum fluorescence emission (F_m_) (Strasser et al., 2000; Giorio et al., 2011), the dark-adapted maximum quantum yield of PSII photochemistry was calculated by the instrument software as F_v_/F_m_ = (F_m_ − F_o_)/F_m_ according to Kitajima and Butler (1975).

### Biomass determinations

Plant leaf area (PLA, m^2^) was measured, at the end of the experiment, on excised leaves using a scanning planimeter (Li-3100, LiCor, Lincoln, NE, U.S.A.). The instrument is equipped with a fluorescent source and a solid state scanning camera to measure the area of leaves as they move through it. For dry weight measurements, leaves and stems were oven-dried at 70°C for 10 days until a stable weight was reached. Both leaf area and total dry weight were measured on three plants.

### Isolation of RNA, cDNA synthesis, and qRT-PCR

Total RNA was extracted from leaf tissues using 1 mL of TRIZOL Reagent (Life Technologies, Carlsbad, CA, USA) per 100 mg of pulverized tissue. The homogenized samples were incubated for 5 min at room temperature and 200 μL of chloroform was added per milliliter of TRIZOL reagent. After centrifugation at 12,000 g for 15 min at 4°C, the upper aqueous phase was removed and 500 μL of 100% isopropanol was added. The samples were incubated at room temperature for 10 min and centrifuged at 12,000 g for 10 min at 4°C. The supernatant was removed and the RNA pellet was washed with 1 mL of 75% ethanol and centrifuged at 7500 g for 5 min at 4°C. The pellet was air dried and dissolved in RNase-free water. RNA quantity was measured spectrophotometrically by NanoDrop ND-1000 Spectrophotometer (NanoDropTechnologies), and integrity was verified on a denaturing MOPS/formaldehyde gel. One microgram of DNase-treated total RNA was reverse transcribed using SuperScript II Reverse Transcriptase and oligo (dT_20_) (Life Technologies, Carlsbad, CA, USA) by incubation at 42°C for 50 min. The reaction was stopped by heat inactivation at 70°C. The complementary DNA was diluted 1:20 and 4.5 μL was used for each qRT-PCR reaction, performed with 6.25 μL of 1X Platinum SYBR Green qPCR SuperMix (Life Technologies, Carlsbad, CA, USA) and 1.75 μL of primer mix (4.28 μM). Primers used are listed in Supplementary Table S1. Preparation of reactions was automated using the Liquid Handler Robot Tecan Freedom Evo and ABI 7900 HT (Applied Biosystems, Foster City, CA, USA) and performed with ABI 7900 HT (Applied Biosystems, Foster City, CA, USA). Cycling conditions were 10 min at 95°C, followed by 40 cycles of 95°C for 15 s and 60°C for 1 min. Three biological replicates with three technical repetitions were tested. Quantification of gene expression was carried out using the 2^−ΔΔCt^ method (Livak and Schmittgen, 2001). Elongation Factor EF1-α was used as an endogenous reference gene (Nicot et al., 2005; Corrado et al., 2007) for the normalization of the expression levels of the target genes. RNA extracted from plants grown in control condition served as calibrator sample for relative quantification of gene expression.

### Proline and ABA content measurements

Leaf samples were collected by excising the leaf at the petiole from three biological replicates. Two technical replicates were performed for each sample. Proline content was determined according to the method of Claussen (2005). Two hundred and fifty milligrams of finely ground leaf tissue were suspended in 1.5 mL of 3% sulfosalicylic acid and filtered through a layer of glass-fiber filter (Macherey-Nagel, Ø 55 mm, Germany). One milliliter of Glacial acetic acid and 1 mL ninhydrin reagent (2.5 g ninhydrin/100 mL of a 6:3:1 solution of glacial acetic acid, distilled water and 85% ortho-phosphoric acid, respectively) were added to 1 mL of the clear filtrate. The mixture was incubated for 1 h in a boiling water bath. The reaction was terminated at room temperature for 5 min. Readings were taken immediately at a wavelength of 546 nm. The proline concentration was determined by comparison with a standard curve.

For ABA measurements, 150 mg of fine powder were extracted in distilled, autoclaved water with constant shaking at 4°C overnight in the dark. The supernatant was collected after centrifugation (10,000 × g for 10 min) and diluted 50-fold with TBS buffer (50 mM TRIS, 1 mM MgCl2, 150 mM NaCl, pH 7.8). Subsequently, ABA was analyzed by indirect enzyme-linked assay (ELISA) using the Phytodetek ABA test kit (Agdia, Elkhart, IN, USA) following the manufacturer's instructions. Color absorbance following reaction with substrate was read at 405 nm using a plate autoreader (1420 Multilabel Counter Victor^3^TM, PerkinElmer).

### Statistical analyses

The statistical significance of soil water content, gas-exchange and fluorescence parameters, ABA and proline contents between water treatments was evaluated through Student's *t*-test.

### RNA library preparation and library sequencing

RNA pools of three biological replicates were used for all RNA-Seq experiments. The total RNA was DNase treated and purified using the RNeasy Plant Mini kit (Qiagen) following manufacturer's protocol (Qiagen, Valencia, CA, USA). RNA samples were analyzed quantitatively and qualitatively by NanoDrop ND-1000 Spectrophotometer (NanoDropTechnologies) and by Bioanalyzer (Agilent Technologies, Santa Clara, CA, USA).

cDNA libraries were prepared with 1 μg of starting total RNA and using the Illumina TruSeq RNA Sample Preparation Kit (Illumina, San Diego, CA), according to TruSeq protocol. Library size and integrity were determined using the Agilent Bioanalyzer 2100 (Santa Clara, CA). Each library was diluted to 2 nM and denatured. Eight pM of each library was loaded onto cBot (Illumina, San Diego CA) for cluster generation with cBot Paired End Cluster Generation Kit (Illumina, San Diego, CA) and sequenced using the Illumina HiSeq 1500 with 100 bp paired-end reads in triplicate obtaining ~14 million reads for replicate. The sequencing service was provided Genomix4life Ltd (http://www.genomix4life.com) at laboratory of Molecular Medicine and Genomics (University of Salerno, Italy).

### RNA sequencing analysis

The cleaning of the raw sequences from the RNA sequencing data was made using the Trim Galore package (http://www.bioinformatics.babraham.ac.uk/projects/trim_galore/). In the first step, low-quality bases were trimmed from the 3′ end of the reads. In the second step, Cutadapt (Martin, 2011) removed adapter sequences; the default settings for paired-end was used. The quality check of the remaining sequences was performed using FastQC (http://www.bioinformatics.babraham.ac.uk/projects/fastqc/). The (1) cleaned pairs, and (2) the high quality single reads obtained after the cleaning step, were used as input for the mapping to the tomato genome (version 2.40), independently. Bowtie version 2.1.0 (Langmead and Salzberg, 2012) and Tophat version 2.0.8 (Kim et al., 2013) were used for mapping. Paired and single reads, uniquely mapped, were counted, independently, per gene available from the iTAG annotation, version 2.3, using the HTSeq-count (http://www-huber.embl.de/users/anders/HTSeq/) version 0.5.4p1, in “union” default mode setting.

In order to define the set of expressed genes, raw read counts were normalized to RPKM (Reads per Kilobase per Million) and genes above the 1 RPKM cut-off were kept for the subsequent analyses. Differential expressed genes (DEGs) were found performing the negative binomial test implemented in the DESeq package (Anders and Huber, 2010) version 1.10.1, at a false discovery rate threshold (FDR) 0.01.

The k-means (MacQueen, 1967) cluster analyses were then performed on the log_2_ of the gene expression level (size factor normalized implemented in *DESeq* Package (Anders and Huber, 2010) for DEGs detected in all the stages, using 20 cluster, a number defined by the Elbow method (Thorndike, 1953), i.e., minimizing the within group variance at different cut-offs. GO enrichments were estimated via the goseq Bioconductor package (Young et al., 2010) (FDR ≤ 0.05) on each detected cluster possessing similar expression profiles in all the stages. As goseq requires gene length data, median transcript length per gene was obtained by parsing with a custom R script cDNA fasta files, as obtained from Ensembl Plants repository. GO annotations for tomato genes were obtained via BLAST2GO (Conesa and Götz, 2008) against NR databases and default settings. Unless otherwise stated, further graphical outputs were obtained with R custom scripts.

## Results

**Figure 1:**
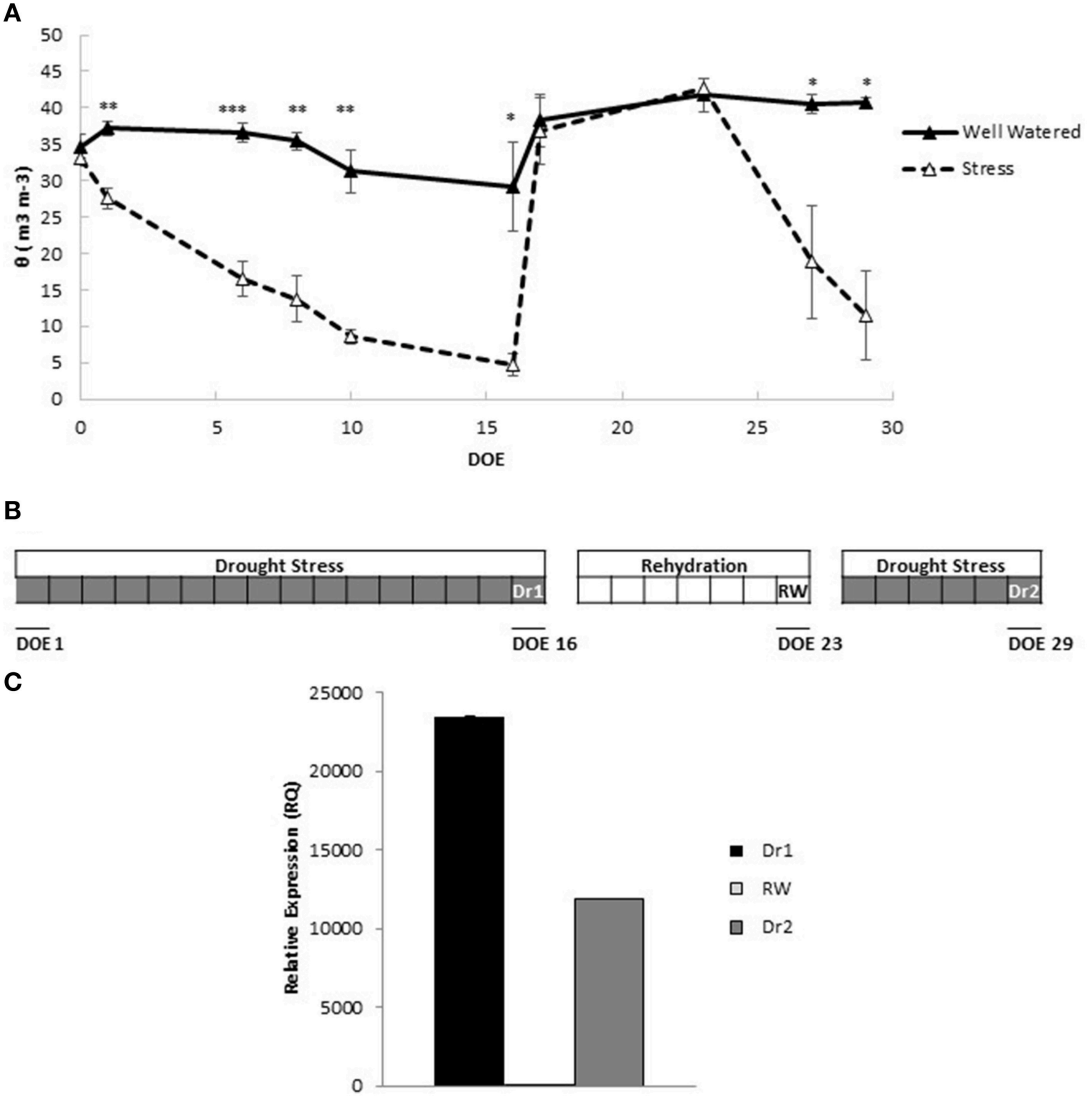
**Experimental outline**. **(A)** Volumetric soil water content (θ) throughout the progression of the experiment. Values represent average measurements ± SD of three replicates. Asterisks denote significant differences according to Student's *t*-test between well watered and stressed pots. ^*^, ^**^, and ^***^ indicate significantly different values in drought stress compared to well-watered pots at *p* ≤ 0.05, *p* ≤ 0.01, and p ≤ 0.001, respectively. **(B)** Schematic representation of the experimental design highlighting the points Dr1 (16 d of irrigation withholding), RW (7 days of irrigation), and Dr2 (6 days of irrigation withhold). **(C)** Gene expression of *Solyc03g116390.2.1* in leaves after two cycles of drought stress (Dr1 and Dr2) and 1 week of rewatering (RW). RNA samples extracted from leaves of well watered plants were used as controls. Gene expression analyses were conducted by qRT-PCR. DOE, days of experiment.

To gain a comprehensive understanding of mechanisms activated by dehydration and rehydration events, we subjected tomato plants to cycles of drought stress and rewatering. Soil water status was monitored throughout the experiment as a measure of the progression of drought stress, and recovery under rewatering. Figure 1A shows values of pots containing plants of the M82 genotype. When water was withheld, pots subjected to drought stress underwent a continuous decline in soil water content (θ), which was significantly lower compared to control pots starting 2 days from the beginning of water withholding (Figure 1A). Drought continued until θ was ~22% of the control pots. This was the maximum stress point (Dr1) for the 1st cycle of drought. Reinstatement of irrigation allowed an immediate full recovery of θ to control values, which were maintained until the end of rewatering (RW). A rapid decline in θ (Figure 1A) was observed when a second cycle of drought stress was imposed and values similar to those of Dr1 were measured 6 days after the beginning of drought treatment (identified as Dr2). A graphic representation of the progression of the experiment is depicted in Figure 1B.

For a preliminary evaluation of the efficacy of the drought stress and rehydration cycles at the cellular level, gene expression of *Solyc03g116390.2.1*, encoding a late embryogenesis abundant protein previously shown to be inducible by drought stress (Gong et al., 2010), was measured at Dr1, RW and Dr2. As shown in Figure 1C, a fold increase in gene expression of at least 10.000 times was observed at Dr1 and Dr2 compared to unstressed control plants. At RW, expression levels in rehydrated plants were comparable to controls, indicating that a full recovery had occurred (Figure 1C).

### Physiological effects of drought stress

**Figure 2:**
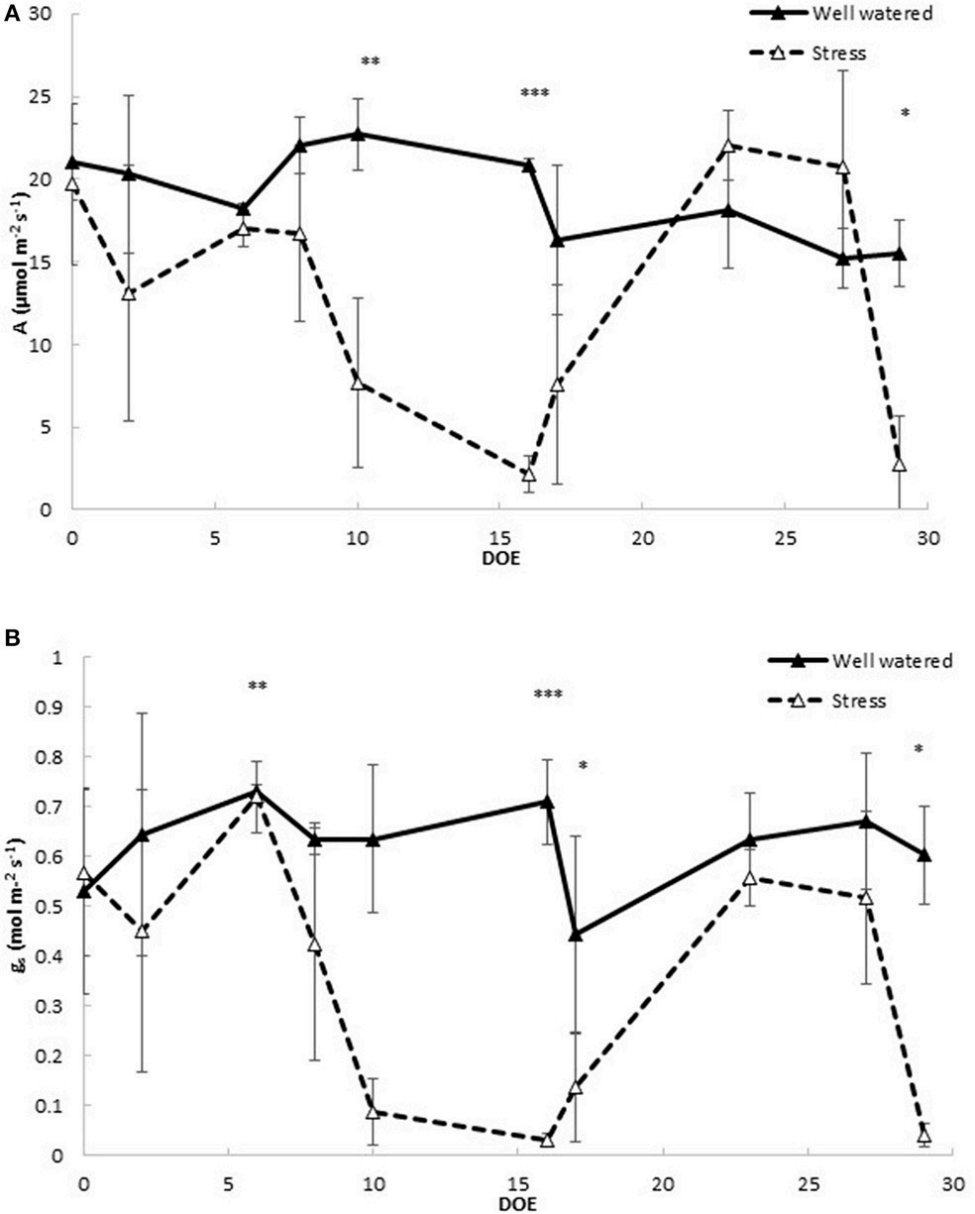
**Leaf gas exchange parameters in well watered and drought stressed plants throughout the experiment**. **(A)** Photosynthetic CO_2_ assimilation (A); **(B)** stomatal conductance to water vapor (gs). Values represent average measurements ± SD, *n* ≥ 5. ^*^, ^**^, and ^***^ indicate significantly different values in drought stressed compared to well-watered plants at *p* ≤ 0.05, *p* ≤ 0.01, and *p* ≤ 0.001, respectively. DOE, days of experiment.

To assess the impact of drought stress and rehydration on the physiology of tomato, leaf gas exchange and photosystem PSII efficiency were measured (Figure 2). Net CO_2_ assimilation rate (Figure 2A) and stomatal conductance to water vapor (Figure 2B) decreased significantly in stressed plants after 10 days of withholding water. At Dr1 CO_2_ assimilation (*A*) decreased to a minimum of 2.2 μmol m^−2^ s^−1^ in the stressed plants, 10% of the CO_2_ assimilation rate measured in controls (Figure 2A). Similar patterns were observed for stomatal conductance (*g*_s_), which in the stressed plants at Dr1 was as low as 0.030, compared to 0.710 mol m^−2^ s^−1^ found in the fully watered plants (Figure 2B). A moderate recovery of both A and g_s_ was observed 1 day after rewatering when soil water content was fully restored (Figure 1A). Both parameters rose to values comparable to those of the controls at RW. Under the second treatment CO_2_ assimilation and stomatal conductance decreased more rapidly as compared to the previous stress cycle, reaching minimum average values of 4.0 μmol m^−2^ s^−1^ for A and 0.070 mol m^−2^ s^−1^ for g_s_ at Dr2 (Figures 2A,B).

**Figure 3:**
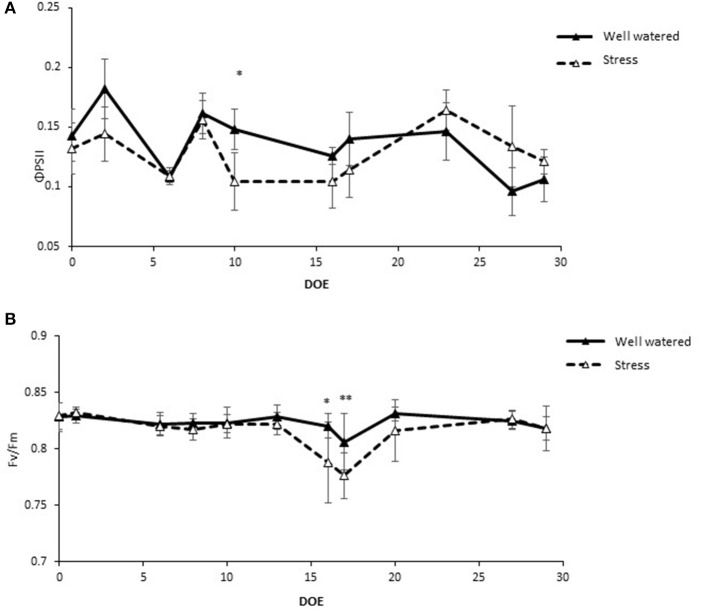
**Chlorophyll fluorescence parameters in well watered and drought stressed plants throughout the experiment**. **(A)** Quantum yield of PSII (ΦPSII); **(B)** Maximum quantum yield of PSII (Fv/Fm). Values represent average measurements ± SD, *n* ≥ 5. ^*^ and ^**^ indicate significantly different values in drought stress compared to well-watered plants at *p* ≤ 0.05 and *p* ≤ 0.01, respectively. DOE, days of experiment.

**Figure 4:**
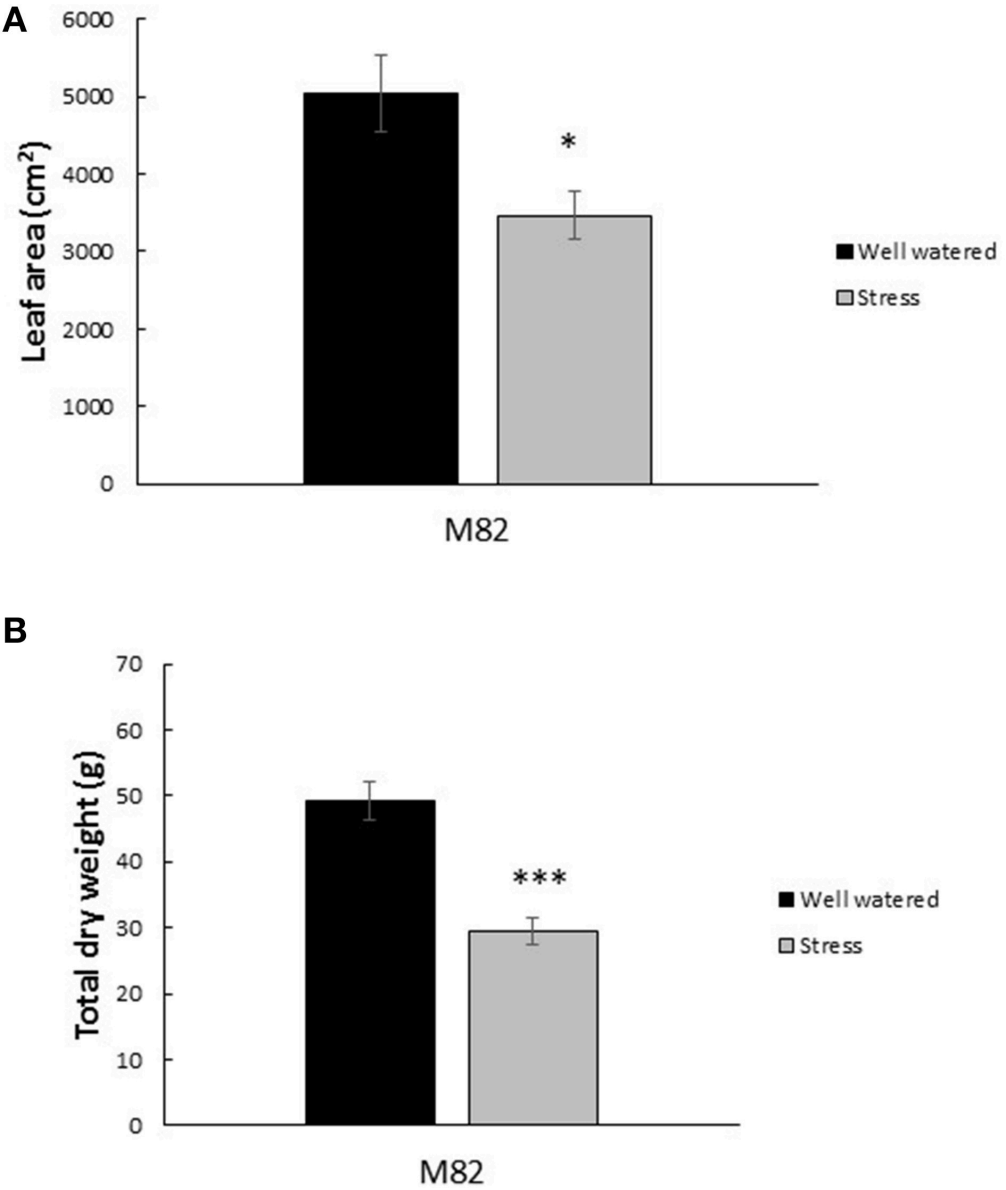
**Impact of drought stress cycles on biometric parameters**. Average Leaf Area **(A)** and total dry weight **(B)** in well watered and drought stress treatments (*n* = 3). For dry weight measurements, roots, stems, leaves, and fruits were included. Measurements were taken at the end of the experiment. ^*^ and ^***^ indicate significantly different values in drought stress compared to well-watered plants at *p* ≤ 0.05 and *p* ≤ 0.001, respectively.

The effective quantum yield of PSII (ϕ_PSII_) decreased in response to the first drought treatment from an average of 0.13 in the control plants to 0.10 in the stressed plants (Figure 3A). Upon rewatering these values recovered to levels similar to controls. These values experienced even steeper declines during the 6 days of the second drought treatment (Figure 3A). The maximal quantum yield of the Photosystem II (PSII), measured in dark-adapted leaves, (F_v_/F_m_) remained stable throughout the experiment in the range of 0.81–0.83 in the control treatments while the values at Dr1 were lower, about 0.77 for F_v_/F_m_. Stressed plants partially recovered after 1 day of rewatering and completely recovered by RW (Figure 3B). Water stress reduced plant leaf area by 31% and dry matter accumulation by an average of 40% (Figures 4A,B).

### Proline and ABA content in drought stressed tomato

**Figure 5:**
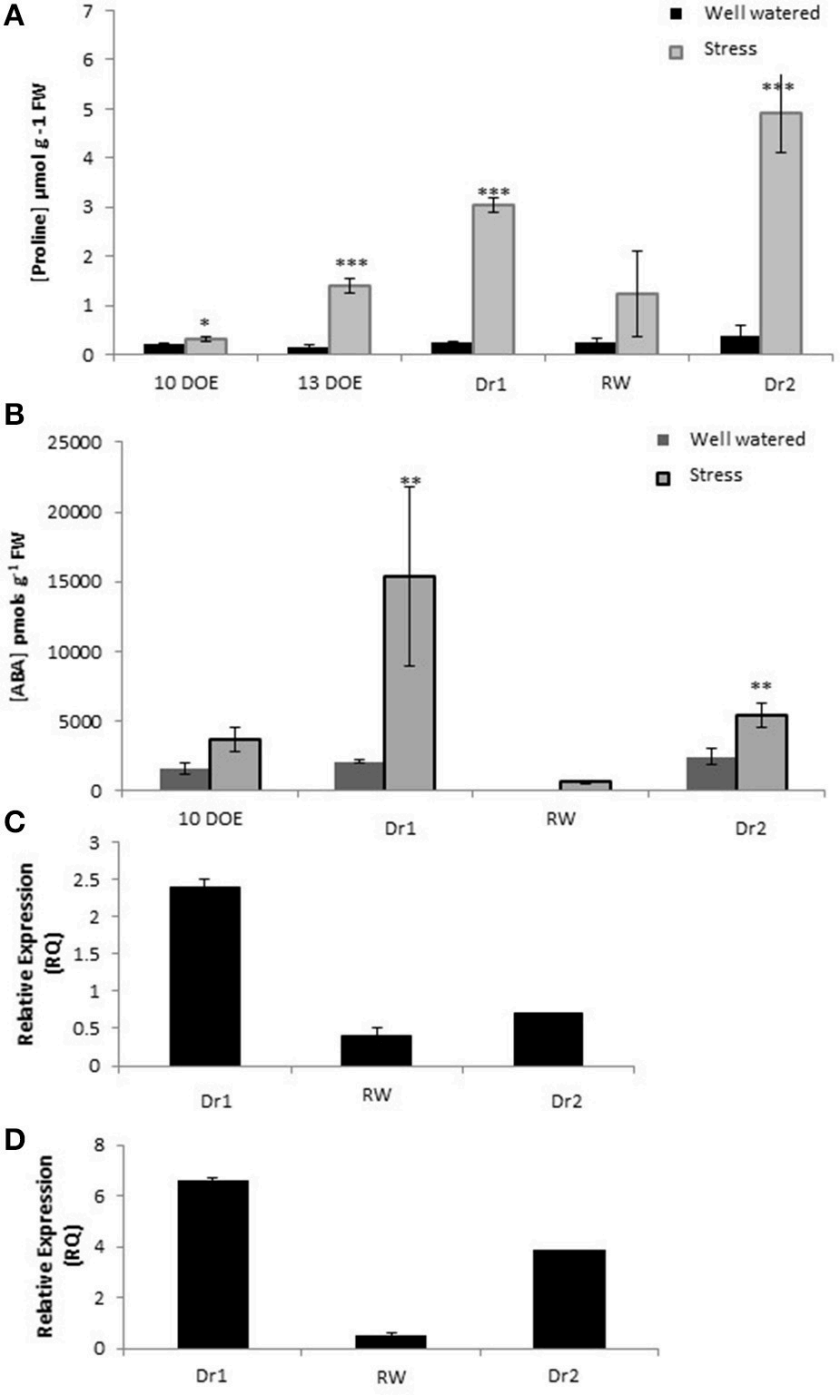
**Quantification of Proline (A) and ABA content (B) in leaves; Gene expression of rate limiting enzymes *P5CS* (C) and *NCED* (D)**. DOE, days of experiment. ^*^, ^**^ and ^***^ indicate significantly different values in drought stressed compared to well-watered plants at *p* ≤ 0.05, *p* ≤ 0.01, and *p* ≤ 0.001, respectively.

Well known metabolic alterations induced by drought stress include leaf accumulation of the osmolyte proline (Claussen, 2005) and the hormone ABA (Sharp and LeNoble, 2002). We therefore measured proline and ABA content at several time points in our experiment by a spectrophotometer and ELISA assay, respectively (Figure 5). After 13 days of drought stress, there was a slight accumulation of proline in the stressed plants. Under more severe stress conditions (Dr1) the amount of proline in the stressed plants was about 10 fold higher than the control M82. Proline amount in the stressed plants decreased in response to rewatering. However, values were still higher than controls for both genotypes at RW. The accumulation of proline reached the highest observed values of 4.5 at Dr2 (Figure 5A).

Ten days of drought stress was not sufficient to elicit accumulation of leaf ABA as compared to the controls (Figure 5A). As the stress became more severe leaf ABA content was as high as 15,367 picomols/g fresh weight at Dr1. After rewatering, ABA levels decreased to values lower than those measured in the control plants at day 10 of experiment. At the end of second drought cycle (6 days of stress, Dr2), ABA content was 10 fold higher than that measured at RW, and about twice the 10-day stress values of first drought period (Figure 5B).

To investigate the correlation between proline and ABA accumulation and transcription of related biosynthetic genes we measured the gene expression of two rate-limiting steps in their biosynthetic processes. Expression of *Solyc08g043170.2.1* and *Solyc07g056570.1.1* encoding a Pyrroline-5-carboxylate synthetase (*P5CS*) and a 9-cis-epoxycaratenoid dioxygenase (*NCED*) respectively was evaluated by qPCR. In Dr1, *P5CS* was induced, while in RW and Dr2 no significant up-regulation was observed (Figure 5C). *NCED* was induced at comparable levels at Dr1 and Dr2, while at RW expression levels were similar to the controls (Figure 5D).

### Transcriptomic perturbations in response to drought stress and rehydration

To identify genes whose expression were altered by drought stress and rewatering in leaves, which could result in the observed physiological alterations, we carried out transcriptome sequencing. We used the Illumina platform on RNA samples extracted from leaves of M82 well-watered plants (WW) as well as in Dr1, RW, and Dr2 (Supplementary Table S2). RNA sequencing derived data were then subjected to gene expression analyses followed by clustering of genes showing similar trends of expression by comparison of the four conditions, and by gene ontology (GO) enrichment analysis. By comparing the different treatments, we identified 966 genes that showed differential expression in at least one of the comparisons, which were therefore considered as Differentially Expressed Genes (DEGs) (Supplementary Table S3).

The analysis highlighted that a large number of DEGs were down-regulated during drought stress. Comparative analysis of drought stressed (Dr1 and Dr2) *vs.* watered plants (WW and RW) revealed 119 DEGs common to all 4 comparisons (Supplementary Table S4). These included several histone encoding genes (e.g., *Solyc10g008910*), cell wall modifying enzymes (e.g., *Solyc04g082140*) as well as heat shock proteins (e.g., *Solyc11g020330*) (Supplementary Table S4).

**Figure 6:**
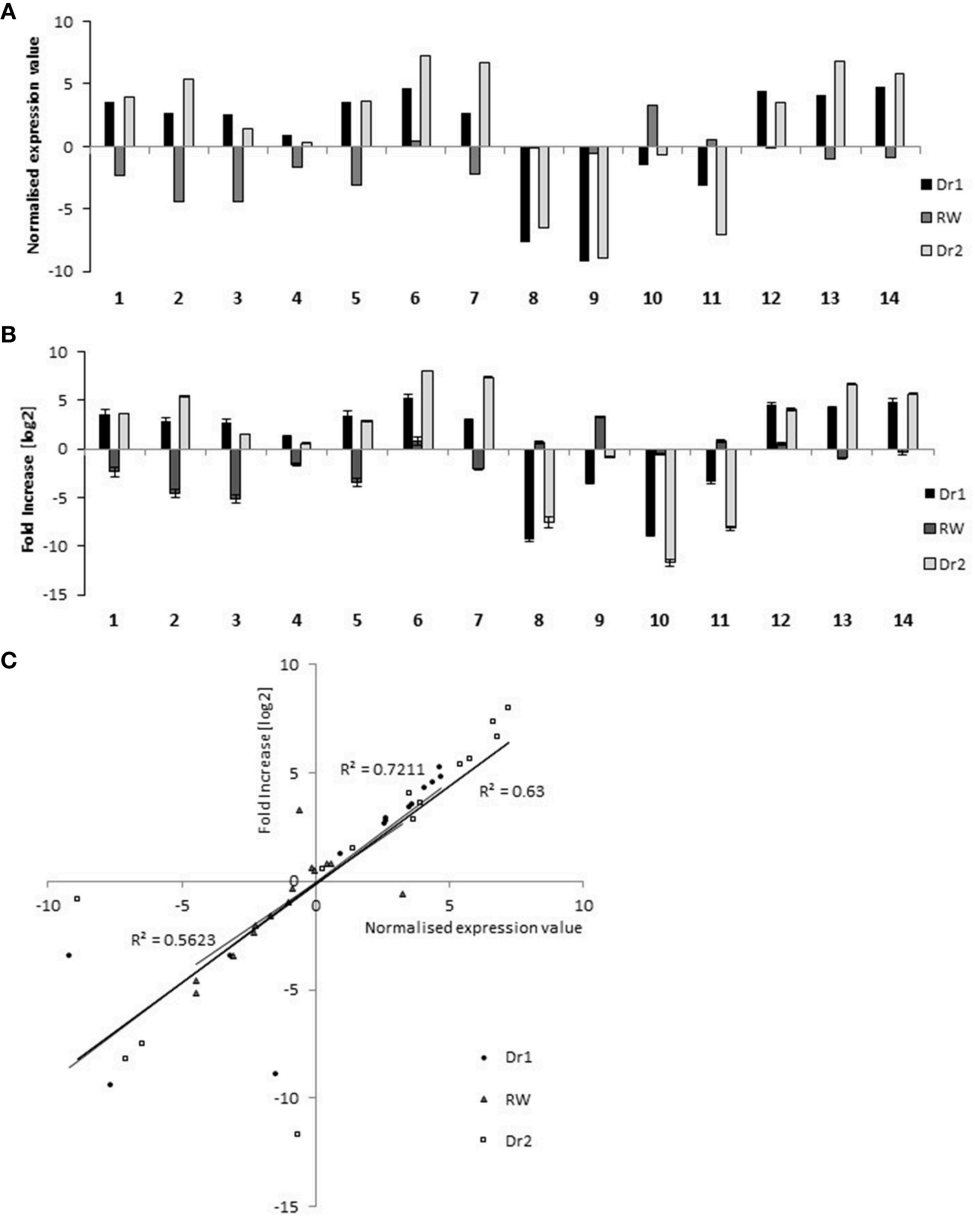
**qRT-PCR validation of RNA sequencing data on 14 selected genes (Supplementary Table S6)**. **(A)** Expression value detected by RNA-seq method. **(B)** Expression analysis conducted by qRT-PCR. Data have been plotted on a log_2_ scale. **(C)** Correlation between RNA-Sequencing and qRT-PCR data. The normalized expression value obtained with RNA sequencing (x axis) were compared to the log_2_ of fold increase by qRT-PCR (y axis). RNA from well-watered control plants was used as calibrator sample.

**Figure 7:**
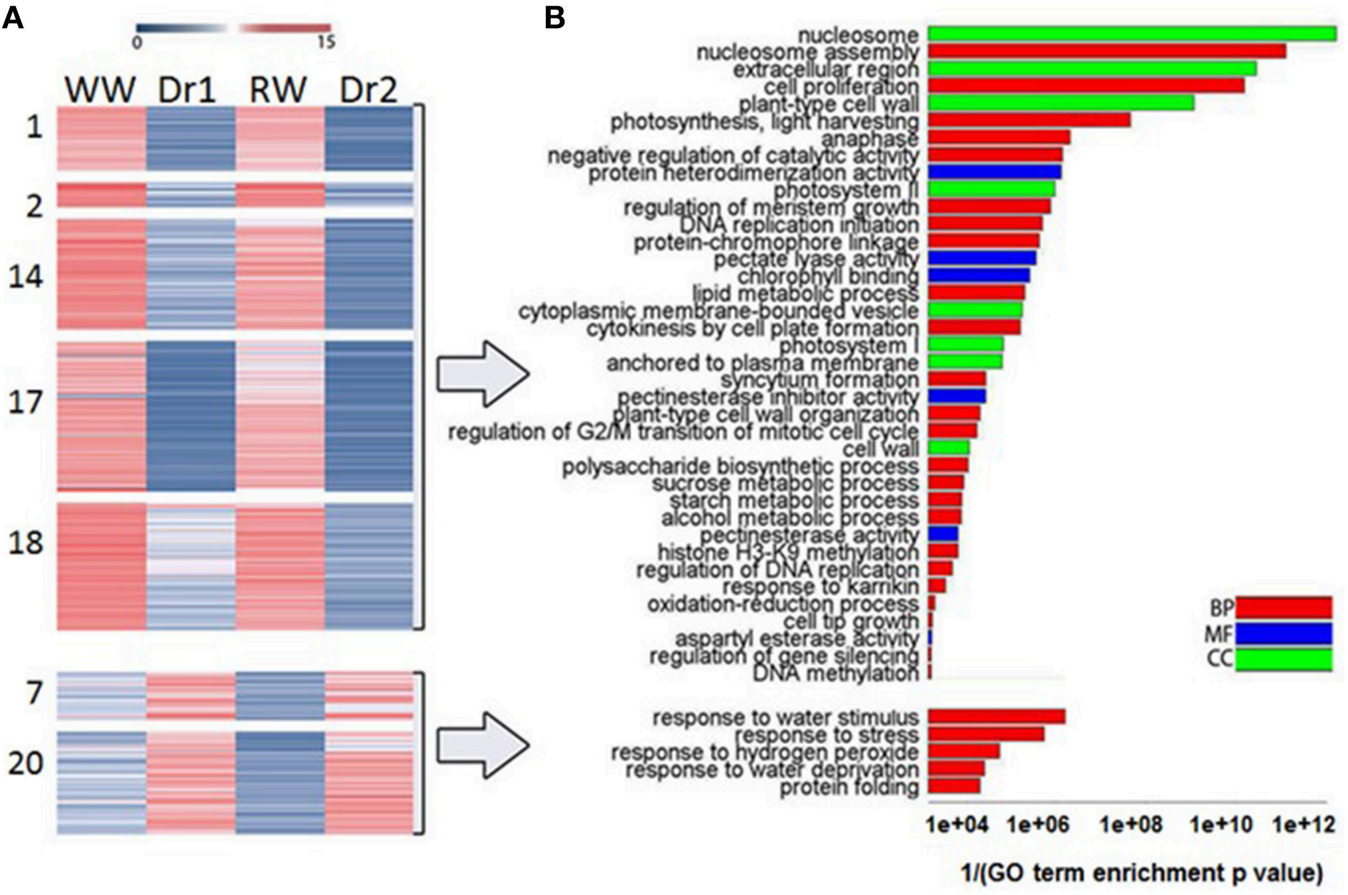
**(A)** Heatmap of selected clusters of Differentially Expressed Genes showing their expression behavior. Red and blue indicate higher and lower expression values, respectively. **(B)** Barplot showing GO Enrichment Analyses (goseq R package, FDR ≤ 0.05) of clusters 1, 2, 14, 17, 18 and 7, 20 independently, plotting GO terms (y axis) and the reciprocal of enrichment p value (x axis). Colors indicate GO ontology: red for Biological Process (BP), blue for Molecular Function (MF), and green for Cellular Component (CC).

In order to classify transcripts based on their behavior in WW, Dr1, RW, and Dr2, a cluster analysis was performed on the DEGs using normalized expression values of the DEGs in each of the experimental conditions. Twenty clusters were identified which grouped transcripts with similar expression trends (Supplementary Table S5). RNA sequencing results and the cluster analysis were validated using qRT-PCR on genes selected from different clusters (Supplementary Table S6, Figure 6). Figures 6A,B show normalized expression values from RNA sequencing and qRT-PCR experiments, respectively. As shown in Figure 6C, a good correlation was observed between the two sets of results. Seven clusters of DEGs were selected for further investigation based on their similar expression patterns (Supplementary Table S5, Figures 7A,B). Among them, clusters 1, 2, 14, 17, and 18 included genes with a higher expression level in WW and RW, while the remaining two clusters 7 and 20 were composed of transcripts with higher expression in Dr1 and Dr2 stressed plants (Figure 7A). Interestingly, clusters containing genes repressed during drought contained several histone variants and chlorophyll binding proteins. Several heat shock proteins and a heat shock factor also appeared to be induced by drought (Table 1). GO enrichment analyses were performed on clusters 1, 2, 14, 17, and 18 and clusters 7 and 20 independently (Figure 7B, Supplementary Tables S7, S8). These analyses showed that genes related to photosynthetic light harvesting (such as Chlorophyll a/b binding protein, *Solyc08g067320*) and to modification of cell wall (i.e., Pectinesterase, *Solyc09g075350*) were down-regulated in Dr1 and Dr2. Several genes encoding sucrose and starch metabolic processes were also down-regulated (Supplementary Table S6).

**Table 1:** **Expression values (Log_2_ RPKM) in WW, Dr1, RW, Dr2 of DEGs belonging to selected functional categories (down, histones, and chlorophyll binding proteins; up, heat shock proteins)**.

**Gene ID**	**Cluster ID**	**Log_2_ RPKM**				**Function**
		**WW**	**Dr1**	**RW**	**Dr2**	
**HISTONES**						
Solyc01g074000.2	14	7.478648295	1.438292852	6.699745948	0.941106311	Histone H3
Solyc01g079110.2	14	7.717402251	1.427606173	6.763411574	0.378511623	Histone H3
Solyc01g080600.2	17	5.459103696	0	4.09592442	0	Histone H3
Solyc01g086820.2	14	7.564835417	1.580145484	6.261530815	0.695993813	Histone H3
Solyc05g054610.1	17	6.787902559	0	5.704318678	0	Histone H4
Solyc06g074790.1	18	8.206526016	3.867896464	7.335211682	2.150559677	Histone H2B
Solyc06g084090.2	17	6.445428759	0.887525271	5.12763328	1.350497247	Histone H2A
Solyc09g074300.1	17	6.513332824	0.650764559	5.369466484	0.704871964	Histone H2A
Solyc10g008910.1	1	8.601436535	2.049630768	7.722807531	1.344828497	Histone H3
Solyc11g072840.1	17	6.533096079	0.35614381	5.329841177	0.687060688	Histone H4
Solyc11g072860.1	1	8.5389261	1.944858446	7.555969495	0.389566812	Histone H4
Solyc11g073260.1	14	6.958378712	3.207892852	5.529508641	1.339137385	Histone H2A
**PHOTOSYSTEM COMPONENTS**						
Solyc08g067330.1	1	9.419538892	2.316145742	9.05058332	0	Chlorophyll a-b binding protein 3C-like
Solyc08g067320.1	1	8.889716892	2.046141782	8.634847344	0	Chlorophyll a/b binding protein
Solyc03g005790.2	1	8.373561216	0.887525271	7.935695463	0.389566812	Chlorophyll a/b-binding protein
Solyc02g070990.1	2	14.18934198	5.337354298	12.9016928	3.370164281	Chlorophyll a/b binding protein
Solyc02g070950.1	2	14.38162474	4.700994494	13.43618319	4.017031081	Chlorophyll a/b binding protein
Solyc03g005780.1	2	14.70360028	3.926948248	14.60010223	3.491853096	Chlorophyll a-b binding protein 3C-like
**HEAT SHOCK-RELATED PROTEINS**						
Solyc12g042830.1	20	2.313245852	6.575766127	1.855989697	6.841218374	Class I heat shock protein
Solyc02g093600.2	20	3.097610797	7.172727518	1.859969548	7.400879436	Class I heat shock protein
Solyc03g113930.1	20	2.313245852	6.969127461	2.720278465	9.552822772	Class IV heat shock protein
Solyc06g053960.2	20	4.286881148	6.571373436	3.261530815	8.040289721	Heat stress transcription factor A3
Solyc09g015000.2	7	9.743252396	12.91458695	7.968263589	13.21605617	Class I heat shock protein
Solyc03g117630.1	7	6.822475232	10.43675318	5.586464526	12.58523251	Heat shock protein
Solyc03g123540.2	7	6.808256325	9.913577595	5.879950768	10.38608881	Class II heat shock protein

A full list of DEGs along with their expression in the four conditions is available in Supplementary Table S5.

GO categories enriched in clusters 7 and 20, instead, were more specifically related to stress, including classes such as response to water stimulus (members included dehydrin, *Solyc01g109920.2*) and water deprivation (including genes such as 2 NAC domain encoding IPR003441- *Solyc12g013620.1/Solyc07g063410.2*) (Figure 7, Supplementary Table S8). Transcripts coding for proteins involved in protein folding (Peptidyl-prolyl cis-trans isomerase *Solyc09g092690.2* and heat shock protein *Solyc03g117630.1*) were also induced by water stress.

## Discussion

In the present study, we have provided a detailed picture of physiological, metabolic and molecular adjustments employed by adult plants of tomato when exposed to events of prolonged water stress. The progression of stress was assessed by detailed monitoring of soil water content and stomatal conductance (g_s_). Leaf water status was strongly impaired by drought stress, as indicated by the very low values of g_s_ observed at Dr1 and Dr2 (Figure 2B). Lutfor Rahman et al. (1999) found in four tomato genotypes that after ca. Ten days of interrupted irrigation, soil water content and g_s_ decreased to values quite similar to our data, and a leaf water potential ranging from −10 to −14 MPa, indicating severe water stress. On this basis, a condition of severe stress can be hypothesized when plants approached Dr1 or Dr2. As expected (Hsiao et al., 1976), both leaf area and dry weight of the entire plant were significantly reduced in response to water deficit (Figure 4). The reduction in photosynthetic rate also likely contributed to plant growth reduction (Figure 2; Chaves et al., 2002). We found that the CO_2_ concentration inside the leaf (data not shown) was not affected during the first week of drought treatment, when soil water deficit (Figure 1) had no clear effect on both A and g_s_ (Figure 2). During subsequent days of stress, both stomatal conductance (Figure 2B) and intercellular [CO_2_] (data not shown) showed a decrease. This indicates the action of stomatal limitation to A, while concomitant non-stomatal limitations, especially during progression to Dr1 should not be ruled out (Tezara et al., 1999; Cifre et al., 2005). The efficiency of the electron transport in the photosystems also decreased as stress progressed (Figure 3). The low F_v_/F_m_ ratio at Dr1 likely indicates that photoinactivation of PSII may have occurred (Baker, 2008). Therefore, we conclude that photochemical limitations contributed to the decline in CO_2_ assimilation along with the altered metabolic content under severe drought (Cifre et al., 2005; Chaves et al., 2009).

These inferences are consistent with the effects observed in response to rewatering. Upon rehydration, a prompt recovery of CO_2_ assimilation in response to stomata reopening would indicate no significant impairment of the photosynthetic machinery (Cornic, 2000).

Chaves et al. (2009) reviewed that a recovery in CO_2_ assimilation of about 50% within a day from rewatering indicates severe water stress and that a few more days are required to reestablish the photosynthetic machinery. Recently, a complete recovery of the photosynthetic rate 24 h after rewatering was observed when mild drought conditions were applied (Nilsen et al., 2014). The moderate recovery observed here in both A and g_s_ after 1 day of soil rewatering is therefore an additional indication of severe drought stress conditions.

One of the goals of this study was it identify potential factors controlling growth under drought stress conditions. At the molecular level, several transcription factors were up-regulated during drought stress, which could also help to explain the observed drought-induced growth reduction, in addition to the induction of stress responses (Supplementary Table S5). We found that two isoforms of subunit A of Nuclear factor Y (NF-Y), encoding orthologs of Arabidopsis *NF-YA7* and *NF-YA10* were also induced by drought stress. NF-Y is a heterotrimetric transcription factor whose subunit A is responsible for binding to DNA promoter sequences containing the CCAAT-box. Over-expression of NF-YAs, including NF-YA7 and NF-YA10, causes a dwarf phenotype and an increase in stress tolerance. Expression of NF-YA transcripts is stress-inducible and is inhibited in *Arabidopsis* in control conditions by miR169, a microRNA present in several isoforms (Li et al., 2008; Leyva-González et al., 2012). The changes in gene expression we observed in NF-Y isoforms likely play a role in growth retardation under the imposed drought conditions.

Our results also yielded other GO categories that contribute to growth and development. GO categories enriched in gene clusters down-regulated by drought stress included the cellular compartment “plant-type cell wall,” “cell wall” and the molecular function “pectate lyase activity” and “pectinesterase activity,” suggesting that cell wall modifying activities are repressed after prolonged drought stress. These categories included genes encoding cell wall modifying enzymes such as two laccase-22 (*Solyc02g065170.2*; *Solyc07g052240.2*), several pectinesterases and four expansins (*Solyc05g007830.2, Solyc06g005560.2, Solyc06g076220.2, Solyc07g054170.2*).

We observed that the tomato heat stress transcription factor *HsfA3* was also up-regulated in drought stress conditions. In *Arabidopsis HsfA3* is regulated by the dehydration-responsive element binding protein 2A (DREB2A). Plants overexpressing DREB2A are drought and heat tolerant and show increased levels of *HsfA3* (Yoshida et al., 2008). *HsfA3* is a powerful driver of heat shock protein expression and probably accounts for the observed up-regulation of several members of the Heat shock protein family, in several species including tomato (Table 1; Supplementary Table S5; Schramm et al., 2008; Li et al., 2013). Over expression of *HSFA4a* and *HSFA9*, two targets of *HsfA3*, has been reported to increase desiccation tolerance in Helianthus (Personat et al., 2014). *HsfA3* may play a role in stress tolerance beyond heat tolerance and merits further study based on our results.

We found the presence of high levels of ABA (Figure 5) and of several targets of the ABA signal transduction pathway among the drought up-regulated genes. Included in these were *RD29B* (*Solyc03g025810.2*) and several LEA proteins indicating that the pathway is active in tomato after prolonged drought stress. The concomitant up-regulation of inhibitors of the ABA signaling cascade such as putative orthologs of Arabidopsis *PP2CA* (Solyc03g096670.2), *MFT* (*SELFPRUINING 2G, Solyc02g079290.2*), and *AFP3* (*Solyc05g012210.2*; Garcia et al., 2008) was also observed. The increased expression of these genes indicates that a negative feedback loop is also in place. This is analogous to reports in *Arabidopsis* plants exposed to moderate drought stress, where the up-regulation of effectors of the ABA response also led to expression of negative regulatory components (Clauw et al., 2015).

Drought stress caused induction of three NAC domain-containing transcription factors (Supplementary Table S5), two of which encode *JA2* (*Solyc12g013620*) and *JA2like* (*JA2L, Solyc07g063140*). These have been recently shown to have antagonistic roles in stomatal movements in tomato during pathogen attack (Du et al., 2014). *JA2* is induced by ABA and promotes stomatal closure through induction of expression of the ABA biosynthetic gene *NCED1.* In contrast, *JA2L* is induced by the bacterial virulence factor coronatine and is proposed to have a role in stomatal reopening by regulating the expression of genes involved in Salicylic acid metabolism (Du et al., 2014). The presence of both *JA2* and *JA2L* in our list of drought-induced genes indicates that these two TFs might also be involved in abiotic stress-triggered stomatal movements and represents a further indication that antagonistic pathways concur to the final balance of physiological adjustments.

The GO Enrichment analysis of the categories over-represented in clusters showing interesting patterns allowed for the identification of specific functions regulated by drought stress (Figure 7, Supplementary Tables S7, S8). Several GO categories related to photosynthesis, such as “photosystem I,” “photosystem II,” “chlorophyll binding,” and “photosynthesis, light harvesting” were enriched in clusters containing genes with higher expression in well-watered rather than drought stressed samples. This possibly indicates a reduced synthesis of components of the photosynthetic machinery under stress conditions (Clusters 1, 2, 14, 17, 18; Table 1). These differences in expression correlated with the low photosynthetic assimilation rate observed in drought stressed plants (Figure 2). A similar down-regulation of photosynthetic genes was observed in a progressive drought stress treatment on Arabidopsis plants. Moderate drought stress reduced the photosynthesis rate while expression of photosynthetic genes was not affected (Harb et al., 2010). This suggests that the mode of drought stress application and the severity of the stress influence the impact on the photosynthetic machinery. Concomitant with the downregulation of components of the photosystem under drought, we observed an upregulation of a FtsH homolog (*Solyc03g112590.2*) with a predicted chloroplast target peptide. FtsHs are ATP-dependent zinc metalloproteases, which, in chloroplasts, have been suggested to be involved in the turnover of the oxidized D1 protein of the PSII reaction center during recovery from photoinhibition (Lindahl et al., 2000; Bailey et al., 2002). Recently, Zhang et al. (2014) observed a progressive and constant upregulation of FtsH in *Medicago truncatula* subjected to drought stress and suggested a role in the repair of PSII damages resulting from drought-induced oxidative stress (Zhang et al., 2014).

Reduction of leaf growth occurs as a result of reduced cell division and/or cell expansion. Analysis of the GO enrichment categories suggests that both cell division and expansion are affected in tomato during drought stress. Categories such as “Cell Proliferation,” “Anaphase,” “Regulation of DNA replication” were enriched in well-watered rather than drought stressed samples, indicating that cell division is very likely repressed during drought.

We also observed that histone s gene families were the most down-regulated group of functional categories (Table 1). Histone expression is regulated during the progression of the cell cycle and tightly connected to DNA replication (Meshi et al., 2000; Rattray and Müller, 2012). The repression of the expression of several H3 and H4 isoforms (Table 1, Supplementary Tables S5, S6), concomitant with repression of genes such as a DNA topoisomerase, two DNA polymerase, single-stranded DNA-binding replication protein A large subunit, single-stranded DNA binding protein p30 could be an indication of a reduction in cell division. This is in all probability one of the factors leading to growth reduction in stress conditions. The observed repression of histone expression under drought is consistent with a recent report in rice showing that expression of several histone isoforms was reduced upon salt and drought treatment (Hu and Lai, 2015).

This study has sought to establish the transcriptomic profile of severe drought treatment in tomato and subsequent recovery with physiological and metabolic measurements. This combined data provides a comprehensive picture of the plant's status under stress and opens avenues to better understand the mechanisms involved in drought tolerance.

Overall, the results presented in this work indicate that drought stress causes the repression of several genes implicated in photosynthesis/light harvesting and plant growth, which was concomitant with a reduction of CO_2_ assimilation, electronic transport efficiency through the photosystems and a growth reduction. In addition, we found several genes up-regulated by drought stress that encoded late effectors and early regulators of ABA signaling. The expression of these genes was in parallel with an increase of ABA and osmolyte concentration as well as closure of stomata. Therefore, gene expression and physiological responses are intimately interconnected. Analysis of these results has yielded interesting candidate genes that could play novel roles in drought tolerance and adaptation.

## Author contributions

MV, AC, MC, RA, PG, GB, and SG conceived the work and designed experiments. PI, PP, GG, CM, RN, HB, CC, PB, GB performed experiments, analyzed the results, contributed figures. PI, MV, RA, PG, GB, SG wrote the paper. All authors reviewed the manuscript.

### Conflict of interest statement

The authors declare that the research was conducted in the absence of any commercial or financial relationships that could be construed as a potential conflict of interest.
